# Supplementary material for: Utilizing a Nordic Crosswalk for Occupational Coding in an Analysis on Occupation-Specific Prolonged Sickness Absence among 7 Million Employees in Denmark, Finland, Norway and Sweden
Source: Int J Environ Res Public Health. 2022 Nov 25;19(23):15674. doi: 10.3390/ijerph192315674 (PMC9737405; doi:10.3390/ijerph192315674)
Supplement: Supplementary file 1 [file ijerph-19-15674-s001.zip › ijerph-2021749-supplementary.pdf]

International Journal of
Environmental Research and Public Health

MDPI

Supplementary Material

Table S1. The ISCO-88 (COM) – DISCO-88 – FISCO-01 – SSYK-96 – STYRK-98 -Nordic crosswalk.

ISCO88 (COM) Title	ISCO-88(COM)	DISCO-88	FISCO-01	SSYK-96	STYRK-98	Nordic Code	Nordic Title
Legislators and senior government officials	1110	1110	1110	1110	1110	1110	Legislators and senior government officials
					1120		
				1120		1120	Senior officials
Senior officials of political party organisations	1141	1141	1141		1141		
Senior officials of employers', workers' and other economic-interest organisations	1142	1142	1142		1142		
Senior officials of humanitarian and other special-interest organisations	1143	1143	1143		1143		
Directors and chief executives	1210	1210	1210	1210	1210	1210	Directors and chief executives
Production and operations managers in agriculture, hunting, forestry and fishing	1221	1221	1221	1221	1221	1221	Production and operations managers in agriculture, hunting, forestry and fishing
Production and operations managers in manufacturing	1222	1222	1222	1222	1222	1222	Production and operations managers in manufacturing
Production and operations managers in construction	1223	1223	1223	1223	1223	1223	Production and operations managers in construction
Production and operations managers in wholesale and retail trade	1224	1224	1224	1224	1224	1224	Production and operations managers in wholesale and retail trade, restaurants and hotels
Production and operations managers in restaurants and hotels	1225	1225	1225				
Production and operations managers in transport, storage and communications	1226	1226	1226		1225	1220	Production and operations managers in other enterprises
Production and operations managers in business services enterprises	1227	1227	1227	1225	1226		
				1226	1227		
				1227	1228		
Production and operations managers in personal care, cleaning and related services	1228	1228	1228	1228	1229	1228	Production and operations managers in personal care, cleaning and related services
Production and operations managers not elsewhere classified	1229	1229	1229	1229		1229	Unspecified production and operations managers
					1220		
Finance and administration managers	1231	1231	1231	1231	1231		

International Journal of
Environmental Research and Public Health
MDPI

Personnel and industrial relations managers	1232	1232	1232	1232	1232	1232	Personnel and industrial relations managers
Sales and marketing managers	1233	1233	1233	1233	1233	1233	Sales and marketing managers
Advertising and public relations managers	1234	1234	1234	1234	1234	1234	Advertising and public relations managers
Supply and distribution managers	1235	1235	1235	1235	1235	1235	Supply and distribution managers
Computing services managers	1236	1236	1236	1236	1236	1236	Computing services managers
Research and development managers	1237	1237	1237	1237	1237	1237	Research and development managers
Other specialist managers not elsewhere classified	1239	1239	1239	1239	1239	1239	Other specialist managers not elsewhere classified
Managers of small enterprises in agriculture, hunting, forestry and fishing	1311	1311	1311	1311	1311	1311	Managers of small enterprises in agriculture, hunting, forestry and fishing
Managers of small enterprises in manufacturing	1312	1312	1312	1312	1312	1312	Managers of small enterprises in manufacturing
Managers of small enterprises in construction	1313	1313	1313	1313	1313	1313	Managers of small enterprises in construction
Managers of small enterprises in wholesale and retail trade	1314	1314	1314	1314	1314	1310	Managers of small enterprises in wholesale and retail trade, restaurants and hotels
Managers of small enterprises of restaurants and hotels	1315	1315	1315		1315		
Managers of small enterprises in transport, storage and communications	1316	1316	1316		1316		
Managers of small enterprises in business services enterprises	1317	1317	1317	1315	1317	1320	Managers in other small enterprises
				1316			
				1317			
Managers of small enterprises in personal care, cleaning and related services	1318	1318	1318	1318	1318	1318	Managers of small enterprises in personal care, cleaning and related services
Managers of small enterprises not elsewhere classified	1319	1319	1319	1319	1319	1319	Managers of unspecified small enterprises
					1310		
Physicists and astronomers	2111	2111	2111	2111	2111	2111	Physicists and astronomers
Meteorologists	2112	2112	2112	2112	2112	2112	Meteorologists
Chemists	2113	2113	2113	2113	2113	2113	Chemists
Geologists and geophysicists	2114	2114	2114	2114	2114	2114	Geologists and geophysicists
Mathematicians and related professionals	2121	2121	2121	2121	2121	2121	Mathematicians and related professionals
Statisticians	2122	2122	2122	2122	2122	2122	Statisticians
					2130		
Computer systems designers, analysts and programmers	2131	2131	2131	2131		2130	Computing professionals
		2132					
Computing professionals not elsewhere classified	2139	2139	2139	2139			
Architects, town and traffic planners	2141	2141	2141	2141	2141	2141	Architects, town and traffic planners
Civil engineers	2142	2142	2142	2142	2142	2142	Civil engineers

International Journal of
Environmental Research and Public Health

MDPI

Electrical engineers	2143	2143	2143	2143	2143	2143	Electrical engineers
Electronics and telecommunications engineers	2144	2144	2144	2144	2144	2144	Electronics and telecommunications engineers
Mechanical engineers	2145	2145	2145	2145	2145	2145	Mechanical engineers
Chemical engineers	2146	2146	2146	2146	2146	2146	Chemical engineers
Mining engineers, metallurgists and related professionals	2147	2147	2147	2147	2147	2147	Mining engineers, metallurgists and related professionals
Cartographers and surveyors	2148	2148	2148	2148	2148	2148	Cartographers and surveyors
Architects, engineers and related professionals not elsewhere classified	2149	2149	2149	2149	2149	2149	Architects, engineers and related professionals not elsewhere classified
Biologists, botanists, zoologists and related professionals	2211	2211	2211	2211	2211	2211	Biologists, botanists, zoologists and related professionals
Pharmacologists, pathologists and related professionals	2212	2212	2212	2212		2212	Pharmacologists, pharmacists, pathologists and related professionals
Pharmacists	2224	2224	2224	2224	2224		
Agronomists and related professionals	2213	2213	2213	2213	2212	2210	Agronomists and related professionals
				2214			
Medical doctors	2221	2221	2221	2221	2221	2221	Medical doctors
Dentists	2222	2222	2222	2222	2222	2222	Dentists
Veterinarians	2223	2223	2223	2223	2223	2223	Veterinarians
Health professionals (except nursing) not elsewhere classified	2229	2229	2229	2229	2225	2229	Unspecified health professionals (except nursing)
Nursing and midwifery professionals	2230	2230	2230		2230	2230	Nursing and midwifery professionals
				2231			
				2232			
				2233			
				2234			
				2235			
				2236			
				3232			
College, university and higher education teaching professionals	2310	2310	2310	2310	2310	2310	College, university and higher education teaching professionals
Secondary education teaching professionals	2320	2320			2320	2320	Secondary education teaching professionals
			2321	2321			
			2322	2322			
			2323	2323			
				2330		2330	

International Journal of
Environmental Research and Public Health

MDPI

Primary education teaching professionals	2331	2331	2331		**3310**		Primary education teaching professionals and associate professionals
Special education teaching professionals	2340	2340	2340	2340	2340	2340	Special education teaching professionals
Education methods specialists	2351	2351	2351	2351	2351	2350	Education methods specialists and school inspectors
School inspectors	2352	2352	2352		2352		
Other teaching professionals not elsewhere classified	2359	2359	2359	2359	2359	2359	Unspecified teaching professionals
Accountants	2411	2411	2411	2411	2511	2411	Accountants
Personnel and careers professionals	2412	2412	2412	2412	2512	2412	Personnel and careers professionals
				2413		2410	Other business professionals
				2414			
Business professionals not elsewhere classified	2419	2419	2419	2419	2519		
				2480			
Lawyers	2421	2421	2421	2421	2521	2421	Lawyers
Judges	2422	2422	2422	2422	2522	2422	Judges
				2423		2429	Unspecified legal professionals
Legal professionals not elsewhere classified	2429	2429	2429	2429	2523		
Archivists and curators	2431	2431	2431	2431	2531	2431	Archivists and curators
Librarians and related information professionals	2432	2432	2432	2432	2532	2432	Librarians and related information professionals
					3493		
Economists	2441	2441	2441	2441	2541	2441	Economists
Sociologists, anthropologists and related professionals	2442	2442	2442	2442	2542	2442	Sociologists, anthropologists and related professionals
Philosophers, historians and political scientists	2443	2443	2443	2443	2543	2443	Philosophers, historians and political scientists
Philologists, translators and interpreters	2444	2444	2444	2444	2544	2444	Philologists, translators and interpreters
Psychologists	2445	2445	2445	2491	2545	2445	Psychologists
				2225			
Social work professionals	2446	2446	2446	2492		2446	Social work professionals
Authors, journalists and other writers	2451	2451	2451	2451	2551	2451	Authors, journalists and other writers
					3491		
Sculptors, painters and related artists	2452	2452	2452	2452	2552	2450	Sculptors, painters and related artists
				2456			
Composers, musicians and singers	2453	2453	2453	2453	2553	2453	Composers, musicians and singers
Choreographers and dancers	2454	2454	2454	2454	2554	2454	Choreographers and dancers
Film, stage and related actors and directors	2455	2455	2455	2455	2555	2455	Film, stage and related actors and directors
Religious professionals	2460	2460	2460	2460	2560	2460	Religious professionals
Public service administrative professionals	2470	2470	2470	2470		2470	Public service administrative professionals

International Journal of
Environmental Research and Public Health

MDPI

					2411		
					2412		
					2413		
					2419		
Chemical and physical science technicians	3111	3111	3111	3111		3110	Chemical, physical science and engeneering technicians
Chemical engineering technicians	3116	3116	3116	3116	3115		
Civil engineering technicians	3112	3112	3112	3112	3111	3112	Civil engineering technicians
Electrical engineering technicians	3113	3113	3113	3113	3112	3113	Electrical engineering technicians
Electronics and telecommunications engineering technicians	3114	3114	3114	3114	3113	3114	Electronics and telecommunications engineering technicians
Mechanical engineering technicians	3115	3115	3115	3115	3114	3115	Mechanical engineering technicians
Mining and metallurgical technicians	3117	3117	3117	3117	3116	3117	Mining and metallurgical technicians
Draughtspersons	3118	3118	3118	3118		3119	Draughtspersons and unspecified physical and engineering science technicians
Physical and engineering science technicians not elsewhere classified	3119	3119	3119	3119	3119		
			3120		3120	3120	Computing associate professionals
Computer assistants	3121	3121		3121			
Computer equipment operators	3122	3122		3122			
Industrial robot controllers	3123	3123					
Photographers and image and sound recording equipment operators	3131	3131	3131	3131	3131	3130	Photographers and image and sound recording equipment operators
				3132			
Broadcasting and telecommunications equipment operators	3132	3132	3132	3133	3132	3132	Broadcasting and telecommunications equipment operators
Medical Equipment Operators	3133	3133		3134		3139	Medical equipment operators and unspecified optical and electronic equipment operators
Optical and electronic equipment operators not elsewhere classified	3139	3139	3139		3139		
Ships' engineers	3141	3141	3141	3141	3141	3141	Ships' engineers
Ships' deck officers and pilots	3142	3142	3142	3142	3142	3142	Ships' deck officers and pilots
Aircraft pilots and related associate professionals	3143	3143	3143	3143	3143	3143	Aircraft pilots and related associate professionals
Air traffic controllers	3144	3144	3144	3144	3144	3140	Air traffic controllers and safety technicians
Air traffic safety technicians	3145	3145	3145	3145			
Building and fire inspectors	3151	3151	3151	3151	3151	3151	Building and fire inspectors
Safety, health and quality inspectors	3152	3152	3152	3152	3152	3152	Safety, health and quality inspectors
Life science technicians	3211	3211	3211	3240	3211	3210	Life science technicians

International Journal of
Environmental Research and Public Health

MDPI

					7450		
Agronomy and forestry technicians	3212	3212	3212	3211	3212	3212	Agronomy and forestry technicians
Farming and forestry advisers	3213	3213	3213	3212	3213	3213	Farming and forestry advisers
Medical Assistants	3221	3221		3221	3221	3220	Medical assistants, hygienists, health and environmental officers
Hygienists, health and environmental officers	3222	3222	3222	3222	3222		
Dieticians and nutritionists	3223	3223	3223	3223	3223	3223	Dieticians and nutritionists
Optometrists and opticians	3224	3224	3224	3224	3224	3224	Optometrists and opticians
Dental assistants	3225	3225	3225	3225	3225	3225	Dental assistants
Physiotherapists and related associate professionals	3226	3226	3226	3226	3226	3226	Physiotherapists and related associate professionals
Veterinary assistants	3227	3227	3227	3227	3227	3227	Veterinary assistants
Pharmaceutical assistants	3228	3228	3228	3228	3228	3228	Pharmaceutical assistants
					5137		
Health associate professionals (except nursing) not elsewhere classified	3229	3229	3229	3229	3229	3229	Health associate professionals (except nursing) not elsewhere classified
Nursing associate professionals	3231	3231	3231	3231	3231	3230	Nursing and midwifery associate professionals
Midwifery associate professionals	3232		3232				
				3233			
				3234	3232		
				3235			
				3239			
Pre-primary education teaching professionals	2332		2332			3320	Pre-primary education teaching professionals and associate professionals
Primary education teaching associate professionals	3310	3310					
Pre-primary education teaching associate professionals	3320	3320		3310	3320		
Special education teaching associate professionals	3330	3330				3330	Special education teaching associate professionals
Other teaching associate professionals	3340	3340	3340	3320		3340	Other teaching associate professionals
					3341		
					3349		
Securities and finance dealers and brokers	3411	3411	3411	3411	3411	3411	Securities and finance dealers and brokers
Insurance representatives	3412	3412	3412	3412	3412	3412	Insurance representatives
Estate agents	3413	3413	3413	3413	3413	3413	Estate agents
Travel consultants and organisers	3414	3414	3414	3414	3414	3414	Travel consultants and organisers
Technical and commercial sales representatives	3415	3415	3415	3415	3415	3415	Technical and commercial sales representatives
					5224		
Buyers	3416	3416	3416	3416	3416	3416	Buyers
Appraisers, valuers and auctioneers	3417	3417	3417	3417	3417	3417	Appraisers, valuers and auctioneers

International Journal of
Environmental Research and Public Health

				3418	3418		
Finance and sales associate professionals not elsewhere classified	3419	3419	3419	3419	3419	3410	Unspecified finance and sales associate professionals
Trade brokers	3421	3421	3421	3421	3421	3421	Trade brokers
Clearing and forwarding agents	3422	3422	3422	3422	3422	3422	Clearing and forwarding agents
Employment agents and labour contractors	3423	3423	3423	3423	3423	3423	Employment agents and labour contractors
Business services agents and trade brokers not elsewhere classified	3429	3429	3429	3429	3429	3429	Business services agents and trade brokers not elsewhere classified
Administrative secretaries and related associate professionals	3431	3431	3431	3431	3431	3431	Administrative secretaries and related associate professionals
Legal and related business associate professionals	3432	3432	3432	3432			
Bookkeepers	3433	3433	3433	3433	3432		
					3433	3430	Other administrative associate professionals
Statistical, mathematical and related associate professionals	3434	3434	3434				
Administrative associate professionals not elsewhere classified	3439	3439					
Customs and border inspectors	3441	3441	3441	3441	3441	3441	Customs and border inspectors
Government tax and excise officials	3442	3442	3442	3442	3442	3442	Government tax and excise officials
Government social benefits officials	3443	3443	3443	3443	3443	3443	Government social benefits officials
Government licensing officials	3444	3444			3444		
Customs, tax and related government associate professionals not elsewhere classified	3449	3449		3449	3449	3440	Other government associate professionals
Police inspectors and detectives	3450	3450	3450			3450	Police inspectors and detectives
Social work associate professionals	3460	3460	3460		3460		
				3461		3460	Social work associate professionals
				3462			
Decorators and commercial designers	3471	3471	3471	3471	3471	3471	Decorators and commercial designers
Radio, television and other announcers	3472	3472	3472	3472	3492	3472	Radio, television and other announcers
Street, night-club and related musicians, singers and dancers	3473	3473	3473	3473	3472	3473	Street, night-club and related musicians, singers and dancers
Clowns, magicians, acrobats and related associate professionals	3474	3474	3474	3474	3473		
					3474	3470	Clowns, magicians, acrobats and related associate professionals
				3476			

International Journal of
Environmental Research and Public Health

MDPI

Athletes, sports persons and related associate professionals	3475	3475	3475	3475	3475	3475	Athletes, sports persons and related associate professionals
Religious associate professionals	3480	3480	3480	3480	3480	3480	Religious associate professionals
Stenographers and typists	4111	4111			4111	4111	Keyboard-operating clerks
Word-processor and related operators	4112	4112	4112				
Data entry operators	4113	4113	4113	4111	4112		
Calculating-machine operators	4114	4114	4114				
Secretaries	4115	4115	4115	4112	4113	4115	Secretaries
				4120		4120	Numerical clerks
Accounting and book-keeping clerks	4121	4121	4121		4121		
Statistical and finance clerks	4122	4122	4122		4114		
					4129		
Stock clerks	4131	4131	4131	4131	4131	4130	Stock and production clerks
Production clerks	4132	4132			4132		
Transport clerks	4133	4133	4133	4132	4133	4133	Transport clerks
Library and filing clerks	4141	4141	4141	4140	4141	4140	Coding, proof-reading, library and related clerks
Coding, proof-reading and related clerks	4143	4143					
Mail carriers and sorting clerks	4142	4142	4142	4150	4142	4142	Mail carriers and sorting clerks
Scribes and related workers	4144					4190	Other office clerks
Other office clerks	4190	4190	4190	4190			
					5225		
Cashiers and ticket clerks	4211	4211	4211	4211	4211	4211	Cashiers and ticket clerks
Tellers and other counter clerks	4212	4212	4212	4212	4212	4212	Tellers and other counter clerks
Bookmakers and croupiers	4213	4213	4213	4213	4213	4213	Bookmakers and croupiers
Pawnbrokers and money-lenders	4214	4214	4214	4214	4214	4214	Pawnbrokers and money-lenders
Debt-collectors and related workers	4215	4215	4215	4215	4215	4215	Debt-collectors and related workers
Travel agency and related clerks	4221	4221	4221	4221	4221	4220	Travel agency and related clerks
				4224			
Receptionists and information clerks	4222	4222	4222	4222	4222	4222	Receptionists and information clerks
Telephone switchboard operators	4223	4223	4223	4223	4223	4223	Telephone switchboard operators
Travel attendants and travel stewards	5111	5111	5111	5111	5111	5111	Travel attendants and travel stewards
Transport conductors	5112	5112	5112	5112	5112	5112	Transport conductors
Travel guides	5113	5113	5113	5113	5113	5113	Travel guides
Housekeepers and related workers	5121	5121	5121	5121	5121	5121	Housekeepers and related workers
Cooks	5122	5122	5122	5122	5122	5122	Cooks

Waiters, waitresses and bartenders	5123	5123	5123	5123	5123	5123	Waiters, waitresses and bartenders
Child-care workers	5131	5131	5131	5131	5131	5131	Child-care workers
					5136		
Institution-based personal care workers	5132	5132	5132	5132	5132	5130	Institution-based personal care workers
				5135	5134		
					5135		
Home-based personal care workers	5133	5133	5133	5133	5133	5133	Home-based personal care workers
				5134		5139	Unspecified personal care and related workers
Personal care and related workers not elsewhere classified	5139	5139	5139	5139	5139		
Hairdressers, barbers, beauticians and related workers	5141	5141	5141	5141	5141	5140	Hairdressers, barbers, beauticians and related workers
					7435		
Companions and valets	5142	5142				5142	Companions and valets
Undertakers and embalmers	5143	5143	5143	5142	5142	5143	Undertakers and embalmers
					5143	5149	Unspecified personal services workers
Other personal services workers not elsewhere classified	5149	5149	5149	5149	5149		
Fire-fighters	5161	5161	5161	5151	5161	5161	Fire-fighters
Police officers	5162	5162	5162	**3450**	**3450**	5162	Police officers
Prison guards	5163	5163	5163	5153	5162	5163	Prison guards
Protective services workers not elsewhere classified	5169	5169	5169	5159	5169	5169	Unspecified protective services workers
				**5152**	**5164**		
Fashion and other models	5210	5210	5210	5210	5210	5210	Fashion and other models
Shop, stall and market salespersons and demonstrators	5220	5220	5220		5221	5220	Shop, stall and market salespersons and demonstrators
				5221	5222		
				5222			
				5223			
Stall and market salespersons	5230			5224			
				5225			
				5226			
Field crop and vegetable growers	6111	6111	6111	6111	6111	6111	Field crop and vegetable growers
Gardeners, horticultural and nursery growers	6112	6112	6112	6112	6112	6112	Gardeners, horticultural and nursery growers
				6113	7129		
Dairy and livestock producers	6121	6121	6121	6121	6121	6121	Dairy and livestock producers
			6122				
Poultry producers	6122	6122		6122	6122	6120	

Animal producers and related workers not elsewhere classified	6129	6129	6129	6129	6129		Poultry and unspecified animal producers and related workers
Crop and animal producers	6130	6130	6130	6130	6130	6130	Crop and animal producers
			6123				
			6140	6140	6210	6140	Motorised farm and forestry plant operators and other forestry and related workers,
Forestry workers and loggers	6141	6141					
Charcoal burners and related workers	6142	6142					
Motorised farm and forestry plant operators	8331	8331	8331	8331			
Aquatic life cultivation workers	6151	6151	6151	6151	6310	6151	Aquatic life cultivation workers
Inland and coastal waters fishery workers	6152	6152	6152	6152	6411	6152	Inland and coastal waters fishery workers
Deep-sea fishery workers	6153	6153				6153	Deep-sea fishery workers
Hunters and trappers	6154	6154	6154	6153	6412	6154	Hunters and trappers
Miners and quarry workers	7111	7111	7111	7111		7110	Mining, mineral-ore and stone-processing-plant operators and related workers
Shotfirers and blasters	7112	7112	7112		7127		
Mining plant operators	8111	8111	8111		8111		
Mineral-ore and stone-processing-plant operators	8112	8112	8112	8111	8112		
Stone splitters, cutters and carvers	7113	7113	7113	7112	7110	7113	Stone splitters, cutters and carvers
Builders	7121	7121	7121			7121	Builders, bricklayers and stonemasons
Bricklayers and stonemasons	7122	7122	7122	7121	7121		
Concrete placers, concrete finishers and related workers	7123	7123	7123	7122	7122	7120	Plasterers, concrete placers, concrete finishers and related workers
Plasterers	7133	7133	7133				
					7123		
					7124		
Carpenters and joiners	7124	7124	7124	7123	7125	7129	Carpenters, joiners and unspecified building frame and related trades workers
					7422		
Building frame and related trades workers not elsewhere classified	7129	7129	7129	7129			
Roofers	7131	7131	7131	7131	7131	7131	Roofers
Floor layers and tile setters	7132	7132	7132	7132		7132	Floor layers and tile setters
Insulation workers	7134	7134	7134	7133	7132	7134	Insulation workers
Glaziers	7135	7135	7135	7134	7133	7135	Glaziers
Plumbers and pipe fitters	7136	7136	7136	7135	7134	7136	Plumbers and pipe fitters
Building and related electricians	7137	7137	7137	7136		7130	Building and related electricians, electrical mechanics fitters and services
Electrical mechanics fitters and services	7241	7241	7241	7241	7241		

International Journal of
Environmental Research and Public Health

Building finishers and related trade workers not elsewhere classified	7139	7139	7139	7139		7139	Unspecified building finishers and related trade workers
Painters and related workers	7141	7141	7141	7141	7141	7140	Painters, varnishers and related workers
Varnishers and Related Painters	7142	7142		7142	7142		
Building structure cleaners	7143	7143	7143	7143		7150	Building structure cleaners and building caretakers
					7143		
					7144		
Building caretakers	9141	9141	9141	7137			
Metal moulders and coremakers	7211	7211	7211	7211	7211	7211	Metal moulders and coremakers
Welders and flame cutters	7212	7212	7212	7212	7212	7212	Welders and flame cutters
Sheet-metal workers	7213	7213	7213	7213	7214	7210	Sheet-metal workers
					7217		
Structural-metal preparers and erectors	7214	7214	7214	7214	7213	7214	Structural-metal preparers and erectors
Riggers and cable splicers	7215	7215	7215	7215	7215	7215	Riggers and cable splicers
Underwater workers	7216	7216	7216	7216	7216	7216	Underwater workers
Blacksmiths, hammer-smiths and forging-press workers	7221	7221	7221	7221	7221	7220	Blacksmiths, tool-makers and related trades workers
					7222		
Tool-makers and related workers	7222	7222	7222	7222			
Machine-tool setters and setter-operators	7223	7223	7223	7223			
Metal wheel-grinders, polishers and tool sharpeners	7224	7224	7224	7224			
Motor vehicle mechanics and fitters	7231	7231	7231	7231	7231	7231	Motor vehicle mechanics and fitters
Aircraft engine mechanics and fitters	7232	7232	7232	7232	7232	7232	Aircraft engine mechanics and fitters
Agricultural- or industrial-machinery mechanics and fitters	7233	7233	7233	7233	7233	7230	Agricultural- or industrial-machinery mechanics and fitters
					7234		
					7235		
					7236		
					7237		
Electronics mechanics, fitters and servicers	7242	7242	7242	7242	7243	7240	Electronics mechanics, fitters and servicers
		7243					
Telegraph and telephone installers and servicers	7244	7244	7244		7242	7250	Telegraph, telephone and electrical line installers and servicers
Electrical line installers, repairers and cable jointers	7245	7245	7245	7243	7244		
Precision-instrument makers and repairers	7311	7311	7311	7311	7311	7311	Precision-instrument makers and repairers
Musical-instrument makers and tuners	7312	7312	7312	7312	7312	7312	Musical-instrument makers and tuners
Jewellery and precious-metal workers	7313	7313	7313	7313	7313	7313	Jewellery and precious-metal workers
Abrasive wheel formers, potters and related workers	7321	7321	7321	7321	7321	7321	Abrasive wheel formers, potters and related workers

International Journal of
Environmental Research and Public Health

MDPI

Glass-makers, cutters, grinders and finishers	7322	7322	7322	7322	7322	7320	Glass and ceramics handicraft workers
Glass engravers and etchers	7323	7323	7323	7323			
Glass, ceramics and related decorative painters	7324	7324	7324	7324	8131		
			7330	7330		7330	Handicraft workers in wood, textile, leather and related materials
Handicraft workers in wood and related materials	7331	7331			7331		
Handicraft workers in textile, leather and related materials	7332	7332			7332		
Compositors, typesetters and related workers	7341	7341	7341	7341	7341	7341	Compositors, typesetters and related workers
					7350		
Stereotypers and electrotypers	7342	7342	7342			7340	Electrotypers, printing, photographic and related workers
Printing engravers and etchers	7343	7343	7343	7342			
Photographic and related workers	7344	7344	7344		7342		
Butchers, fishmongers and related food preparers	7411	7411	7411	7411	7411	7411	Butchers, fishmongers and related food preparers
Bakers, pastry-cooks and confectionery makers	7412	7412	7412	7412	7412	7412	Bakers, pastry-cooks and confectionery makers
Food and beverage tasters and graders	7415	7415	7415	7413	7413	7415	Food and beverage tasters and graders
Wood treaters	7421	7421	7421			7420	Wood treaters, cabinetmakers, woodworking machine setters and setter-operators
Cabinetmakers and related workers	7422	7422	7422	7421	7421		
Woodworking machine setters and setter-operators	7423	7423	7423				
Basketry weavers, brush makers and related workers	7424	7424	7424	7422	7423	7424	Basketry weavers, brush makers and related workers
Tailors, dressmakers and hatters	7433	7433	7433	7431	7432	7433	Tailors, dressmakers and hatters
Furriers and related workers	7434	7434	7434	7432	7433	7434	Furriers and related workers
Textile, leather and related pattern-makers and cutters	7435	7435	7435	7433	7434	7430	Textile, leather and related pattern-makers and cutters
					7443		
Sewers, embroiderers and related workers	7436	7436	7436	7434		7436	Sewers, embroiderers and related workers
Upholsterers and related workers	7437	7437	7437	7435	7436	7437	Upholsterers and related workers
Pelt dressers, tanners and fellmongers	7441	7441	7441	7441	7441	7440	Pelt dressers, tanners and fellmongers
Shoe-makers and related workers	7442	7442	7442	7442	7442		
					8113	8113	Well drillers and borers, and related workers
Well drillers and borers and related workers	8113	8113	8113	8112	8114		
Ore and metal furnace operators	8121	8121	8121	8121	8121	8120	Metal-processing-plant operators
Metal melters, casters and rolling-mill operators	8122	8122	8122	8122	8122		
				8125			
Metal heat-treating-plant operators	8123	8123	8123	8123			
Metal drawers and extruders	8124	8124	8124	8124			
				8130		8130	

International Journal of
Environmental Research and Public Health

MDPI

Glass and ceramics kiln and related machine operators	8131	8131	8131		8132		Glass, ceramics, paper and related machine and plant operators
Glass, ceramics and related plant operators not elsewhere classified	8139	8139	8139		8139		
Paper-pulp plant operators	8142	8142	8142	8143	8142		
Papermaking-plant operators	8143	8143	8143	8144			
			8150	8150		8150	Chemical-processing-plant operators
Crushing-, grinding- and chemical-mixing-machinery operators	8151	8151					
Chemical-heat-treating-plant operators	8152	8152					
Chemical-filtering- and separating-equipment operators	8153	8153					
Chemical-still and reactor operators (except petroleum and natural gas)	8154	8154					
Petroleum- and natural-gas-refining-plant operators	8155	8155			8151		
Chemical-processing-plant operators not elsewhere classified	8159	8159			8159		
				8160		8160	Power-production and related plant operators
Power-production plant operators	8161	8161	8161		8161		
Steam-engine and boiler operators	8162	8162	8162				
Incinerator, water-treatment and related plant operators	8163	8163	8163		8162		
Industrial robot operators	8170	8170	8170	8170		8170	Industrial robot operators
Machine-tool operators	8211	8211	8211	8211	8212	8210	Machine-tool operators
					8213		
Cement and other mineral products machine operators	8212	8212	8212	8212	8214	8212	Cement and other mineral products machine operators
Pharmaceutical-and toiletry-products machine operators	8221	8221	8221	8221	8221	8221	Pharmaceutical-and toiletry-products machine operators
Ammunition- and explosive-products machine operators	8222	8222	8222	8222	8222	8222	Ammunition- and explosive-products machine operators
Metal finishing-, plating- and coating-machine operators	8223	8223	8223	8223	**8225**	8223	Metal finishing-, plating- and coating-machine operators
Photographic-products machine operators	8224	8224	8224	8224	**8254**	8224	Photographic-products machine operators
Chemical-products machine operators not elsewhere classified	8229	8229	8229	8229	8229	8229	Unspecified chemical-products machine operators
Rubber-products machine operators	8231	8231	8231	8231	8223	8231	Rubber-products machine operators
Plastic-products machine operators	8232	8232	8232	8232	8224	8232	Plastic-products machine operators

International Journal of
Environmental Research and Public Health

Wood-products machine operators	8240	8240	8240	8240		8240	Wood-products machine operators
Wood-processing-plant operators	8141	8141	8141	8141	8141		
				8142	8143		
Printing-machine operators	8251	8251	8251	8251	8251	8250	Printing-machine operators and craft textile printers
Silk-screen, block and craft textile printers	7346	7346	7346	7344			
Book-binding-machine operators	8252	8252	8252	8252	8252	8259	Book-binding-machine operators and related workers
Bookbinders and related workers	7345	7345	7345	7343			
Paper-products machine operators	8253	8253	8253	8253	8253	8253	Paper-products machine operators
Fibre-preparing-, spinning- and winding-machine operators	8261	8261	8261	8261	8261	8261	Fibre-preparing-, spinning- and winding-machine operators and related workers
Fibre preparers	7431	7431	7431				
Weaving- and knitting-machine operators	8262	8262	8262	8262	8262	8262	Weaving- and knitting-machine operators and related workers
Weavers, knitters and related workers	7432	7432	7432		7431		
Sewing-machine operators	8263	8263	8263	8263	8263	8263	Sewing-machine operators
Bleaching-, dyeing- and cleaning-machine operators	8264	8264	8264	8264	8266	8264	Bleaching-, dyeing- and cleaning-machine operators
Shoemaking- and related machine operators	8266	8266	8266	8265	8267	8266	Shoemaking- and related machine operators
Fur- and leather-preparing-machine operators	8265	8265	8265			8269	Textile, fur- and leather-preparing-and product machine operators
Textile-, fur- and leather-products machine operators not elsewhere classified	8269	8269	8269	8269	8269		
					8264		
					8265		
Meat- and fish-processing-machine operators	8271	8271	8271	8271		8271	Meat- and fish-processing-machine operators
					8271		
					8272		
Dairy-products machine operators	8272	8272	8272	8272	8273	8272	Dairy-products machine operators and workers
Dairy-products workers	7413	7413	7413				
Grain- and spice-milling-machine operators	8273	8273	8273	8273	8274	8273	Grain- and spice-milling-machine operators
Baked-goods, cereal- and chocolate-products machine operators	8274	8274	8274	8274	8275	8274	Baked-goods, cereal- and chocolate-products machine operators
Fruit-, vegetable- and nut-processing-machine operators	8275	8275	8275	8275	8276	8275	Fruit-, vegetable- and nut-processing-machine operatorsand workers
Fruit, vegetable and related preservers	7414	7414	7414				
Brewers, wine and other beverage machine operators	8278	8278	8278	8278	8277	8278	Brewers, wine and other beverage machine operators
Sugar production machine operators	8276	8276	8276	8276		8270	Sugar, tee, coffee and tobaco production machine operators
Tea-, coffee- and cocoa-processing-machine operators	8277	8277	8277	8277			
Tobacco production machine operators	8279	8279	8279	8279			

International Journal of
Environmental Research and Public Health

MDPI

Tobacco preparers and tobacco products makers	7416	7416	7416				
					8279		
					7419		
Mechanical-machinery assemblers	8281	8281	8281	8281	8281		
Electrical-equipment assemblers	8282	8282	8282	8282			
Electronic-equipment assemblers	8283	8283	8283		8282		
Metal-, rubber- and plastic-products assemblers	8284	8284	8284	8283	8211	8280	Assemblers
Wood and related products assemblers	8285	8285	8285	8284			
Paperboard, textile and related products assemblers	8286	8286	8286	8225			
Composite products assemblers	8287	8287					
Other machine operators not elsewhere classified	8290	8290	8290	8290		8290	Unspecified machine operators
Locomotive engine drivers	8311	8311	8311	8311	8311	8311	Locomotive engine drivers
Railway brakers, signallers and shunters	8312	8312	8312	8312	8312	8312	Railway brakers, signallers and shunters
Motorcycle drivers	8321	8321	8321			8320	Car, taxi, van and motorcycle drivers
Car, taxi and van drivers	8322	8322	8322	8321	8321		
Bus and tram drivers	8323	8323	8323	8322	8322	8323	Bus and tram drivers
Heavy truck and lorry drivers	8324	8324	8324	8323	8323	8324	Heavy truck and lorry drivers
Earth-moving and related plant operators	8332	8332	8332	8332	8331	8332	Earth-moving and related plant operators
Crane, hoist and related plant operators	8333	8333	8333	8333	8332	8333	Crane, hoist and related plant operators
Lifting-truck operators	8334	8334	8334	8334	8333	8334	Lifting-truck operators
Ships' deck crews and related workers	8340	8340	8340	8340			
					8341	8340	Ships' deck crews and related workers
					8342		
Street vendors	9111		9111	9110			
Door-to-door and telephone salespersons	9113	9113	9113	5227	5223	9110	Street vendors and street service elementary occupations
Shoe cleaning and other street services elementary occupations	9120	9120	9120		9120		
				9130			
Domestic helpers and cleaners	9131	9131	9131	9121	9131		
Helpers and cleaners in offices, hotels and other establishments	9132	9132	9132	9122	9132	9130	Domestic and related helpers, cleaners and launderers
Hand-launderers and pressers	9133	9133	9133				
					9133		
Vehicle, window and related cleaners	9142	9142	9142	9123	9141	9140	Vehicle, window and related cleaners

International Journal of
Environmental Research and Public Health

					9142		
					5163		
				9190			
Messengers, package and luggage porters and deliverers	9151	9151	9151	9141	9151	9151	Messengers, package and luggage porters and deliv-erers
Doorkeepers, watchpersons and related workers	9152	9152	9152	9142	9152	9152	Doorkeepers, watchpersons and related workers
Vending-machine money collectors, meter readers and re-lated workers	9153	9153	9153	9143	9153	9153	Vending-machine money collectors, meter readers and related workers
					9160		
Garbage collectors	9161	9161	9161	9150		9160	Garbage collectors and related labourers
Sweepers and related labourers	9162	9162	9162				
			9210	9210	9210		
Farm-hands and labourers	9211	9211				9210	Agricultural, fishery and related labourers
Forestry labourers	9212	9212					
Fishery, hunting and trapping labourers	9213	9213					
				9310	9310		
Mining and quarrying labourers	9311	9311	9311				
Construction and maintenance labourers: roads, dams and similar constructions	9312	9312	9312			9310	Mining and construction labourers
Building construction labourers	9313	9313	9313				
				7124	7126		
					7128		
Manufacturing labourers	9320	9320	9320	9320	9320	9320	Manufacturing labourers
Transport labourers and freight handlers	9330	9330	9330	9330	9330	9330	Transport labourers and freight handlers

No matching occupation could be found in the national classification
Nordic code is not applicable for at least one national classification
The same Nordic code was assigned to several occupational groups from the national classifications

International Journal of
Environmental Research and Public Health

MDPI

Table S2. Aggregated occupational groups.

Merged Group Code	Suggested Name	Name in the Nordic Crosswalk	Nordic Code
0001	Managers in computing services, research and development	Computing services managers	1236
		Research and development managers	1237
0002	Managers of wholesale and retail trade and restaurants and hotels	Managers of small enterprises in wholesale and retail trade; restaurants and hotels	1310
		Production and operations managers in wholesale and retail trade, and restaurants and hotels	1224
0003	Managers of small enterprises in agriculture, hunting, forestry and fishing, and growers	Managers of small enterprises in agriculture, hunting, forestry and fishing	1311
		Field crop and vegetable growers	6111
		Gardeners, horticultural and nursery growers	6112
		Aquatic life cultivation workers	6151
		Inland and coastal waters fishery workers	6152
		Hunters and trappers	6154
0004	Managers in manufacturing	Managers of small enterprises in manufacturing	1312
		Production and operations managers in manufacturing	1222
0005	Managers in construction	Managers of small enterprises in construction	1313
		Production and operations managers in construction	1223
0006	Managers in personal care, cleaning and related services	Managers of small enterprises in personal care, cleaning and related services	1318
		Production and operations managers in personal care, cleaning and related services	1228
0007	Managers in storage and communication; business services enterprises, supply and distribution	Managers of small enterprises in storage and communications; business services enterprises	1320
		Production and operations managers in storage and communications business services enterprises	1220
		Supply and distribution managers	1235
0008	Managers in unspecified enterprises	Production and operations managers not elsewhere classified	1229
		Managers of small enterprises not elsewhere classified	1319
0032	Physicists, astronomers and meteorologists	Physicists and astronomers	2111
		Meteorologists	2112
0033	Mathematicians, statisticians and related professionals	Mathematicians and related professionals	2121
		Statisticians	2122
0034	Lawyers, judges and other legal professionals	Lawyers	2421
		Judges	2422
		Legal professionals not elsewhere classified	2429
0035	Professionals and associate professionals in social work	Social work professionals	2446
		Social work associate professionals	3460
0036	Safety and quality inspectors	Building and fire inspectors	3151
		Safety, health and quality inspectors	3152

International Journal of
Environmental Research and Public Health

0037	Agronomy and forestry advisers and technicians	Agronomy and forestry technicians	3212
		Farming and forestry advisers	3213
0010	Dieticians and nutritionists; optometrist and opticians	Dieticians and nutritionists	3223
		Optometrists and opticians	3224
		Unspecified health associate professionals (except nursing)	3229
0011	Nursing and midwifery professionals and associate professionals	Nursing and midwifery professionals	2230
		Nursing and midwifery associate professionals	3230
0012	Education methods specialists and inspectors, and unspecified teaching professionals	Education methods specialists and inspectors	2350
		Other teaching professionals not elsewhere classified	2359
0013	Personnel and careers professionals; employment agents, labour contractors	Personnel and careers professionals	2412
		Employment agents and labour contractors	3423
0014	Social scientists, philosophers and historians	Sociologists, anthropologists and related professionals	2442
		Philosophers, historians and political scientists	2443
0009	Translators and interpreters; journalists and other writers; radio and other announcers	Philologists, translators and interpreters	2444
		Authors, journalists and other writers	2451
		Radio, television and other announcers	3472
0015	Physiotherapists and unspecified health professionals	Health professionals (except nursing) not elsewhere classified	2229
		Physiotherapists and related associate professionals	3226
0038	Customs, tax and related government associate professionals	Government licensing officials, customs, tax and other government associate professionals	3440
		Customs and border inspectors	3441
		Government tax and excise officials	3442
		Government social benefits officials	3443
0016	Travel consultants and travel agency clerks	Travel consultants and organisers	3414
		Travel agency and related clerks	4220
0017	Appraisers, auctioneers and unspecified associate professionals in finance and sales	Finance and sales associate professionals not elsewhere classified	3410
		Appraisers, valuers and auctioneers	3417
0018	Choreographers, dancers, creative and performing artists	Choreographers and dancers	2454
		Clowns, magicians, acrobats and related associate professionals	3470
		Street, night-club and related musicians, singers and dancers	3473
0019	Sculptors, painters and commercial designers	Decorators and commercial designers	3471
		Sculptors, painters and related artists	2450
0039	Data processors and secretaries	Word-processor, data-processing and related operators	4111
		Secretaries	4115
		Coding, proof-reading and related clerks	4140
0020	Cashiers, tellers and other counter clerks	Cashiers and ticket clerks	4211

International Journal of
Environmental Research and Public Health

MDPI

		Tellers and other counter clerks	4212
		Bookmakers and croupiers	4213
		Pawnbrokers and money-lenders	4214
		Debt-collectors and related workers	4215
0021	Travel attendants and guides	Travel attendants and travel stewards	5111
		Travel guides	5113
0040	Unspecified personal services workers	Undertakers and embalmers	5143
		Other personal services workers not elsewhere classified	5149
0022	Animal producers	Poultry and animal producers	6120
		Dairy and livestock producers	6121
0023	Miners, shotfirers, stone cutters, and related plant operators	Miners, shotfirers and mining-, mineral and stone plant operators	7110
		Stone splitters, cutters and carvers	7113
0024	Precision and musical instrument makers, jewellery, glass and handicraft workers	Precision-instrument makers and repairers	7311
		Musical-instrument makers and tuners	7312
		Jewellery and precious-metal workers	7313
		Glass-makers and related trades workers	7320
		Handicraft workers in wood, textile, leather and related materials	7330
		Abrasive wheel formers, potters and related workers	7321
0025	Tailors, sewers and other textile workers	Tailors, dressmakers and hatters	7433
		Sewers, embroiderers and related workers	7436
		Upholsterers and related workers	7437
		Textile, leather and related pattern-makers and cutters	7430
		Furriers and related workers	7434
0026	Metal- and mineral-products machine operators	Metal machine-tool operators	8210
		Cement and other mineral products machine operators	8212
0027	Chemical-products machine operators	Pharmaceutical-and toiletry-products machine operators	8221
		Ammunition- and explosive-products machine operators	8222
		Metal finishing-, plating- and coating-machine operators	8223
		Chemical-products machine operators not elsewhere classified	8229
0028	Rubber- and plastic-products machine operators	Rubber-products machine operators	8231
		Plastic-products machine operators	8232
0029	Book- and paper- products machine operators	Paper-products machine operators	8253
		Book-binding-machine operators and related workers	8259
0041		Printing engravers, photographic and related workers	7340

International Journal of
Environmental Research and Public Health

MDPI

	Photographic and printing workers and machine operators	Textile printers and related machine operators	8250
		Photographic-products machine operators	8224
0030	Agricultural or industrial machinery fitters, and mechanical and electrical equipment assemblers	Agricultural- or industrial-machinery mechanics and fitters	7230
		Motor vehicle mechanics and fitters	7231
		Aircraft engine mechanics and fitters	7232
		Mechanical-machinery and electrical-equipment assemblers	8280
		Other machine operators not elsewhere classified	8290
0042	Bakers and confectionery makers and food and beverage tasters	Bakers, pastry-cooks and confectionery makers	7412
		Food and beverage tasters and graders	7415
0043	Machine operators of textile, fur and leather	Fibre preparing-, spinning- and winding-machine operators	8261
		Weaving- and knitting-machine operators	8262
		Sewing-machine operators	8263
		Bleaching-, dyeing- and cleaning-machine operators	8264
		Shoemaking- and related machine operators	8266
		Textile-, Fur- and leather--machine operators	8269
0044	Grocery and beverage machine operators	Sugar, tea-, coffee and tobacco production machine operators	8270
		Grain- and spice-milling-machine operators	8273
		Baked-goods, cereal- and chocolate-products machine operators	8274
		Fruit-, vegetable- and nut-processing-machine operators and preservers	8275
		Brewers, wine and other beverage machine operators	8278
0031	Watchpersons, meter readers and related workers	Doorkeepers, watchpersons and related workers	9152
		Vending-machine money collectors, meter readers and related workers	9153

International Journal of
Environmental Research and Public Health

MDPI

Legends for Tables S3–S5.

	DK	FI	NO	SE
Incidence of SA does not statistically significantly differ from 0 (95% CI includes 0)				
Incidence of SA statistically significantly differs from 0, but it is lower than population average				
Incidence of SA statistically significantly higher than population average				
Occupational group was excluded due to small size (< 100 persons per occupation)	-	-	-	-

DK- Denmark; FI- Finland; NO- Norway; SE-Sweden;

Major occupation: 1 – Legislators, senior officials and managers; 2- Professionals; 3-Associate professionals; 4- Clerks; 5- Service and care workers, and shop and market sales workers; 6- Skilled agricultural and fishery workers; 7- Craft and related trades workers; 8- Plant and machine operators and assemblers; 9- Elementary occupations.

Table S3. Heatmap of occupational differences in the age-adjusted one-year cumulative incidence of prolonged all-cause SA across four Nordic countries among men and women.

Code	Major Occupation	Title	Men				Women			
			DK	FI	NO	SE	DK	FI	NO	SE
0001	1	Managers in computing services, research and development								
0002	1	Managers of wholesale and retail trade and restaurants and hotels								
0004	1	Managers in manufacturing								
0005	1	Managers in construction								
0006	1	Managers in personal care, cleaning and related services		-				-		
0007	1	Managers in storage and communication; business services enterprises, supply and distribution								
0008	1	Managers in unspecified enterprises								
1110	1	Legislators and senior government officials								
1120	1	Senior officials of special-interest organizations								
1210	1	Directors and chief executives								
1221	1	Production and operations managers in agriculture, hunting, forestry and fishing					-	-		-
1231	1	Finance and administration managers								
1232	1	Personnel and industrial relations managers								
1233	1	Sales and marketing managers								

International Journal of *Environmental Research and Public Health*

1234	1	Advertising and public relations managers								
1239	1	Other unspecified specialist managers								
0009	2	Translators and interpreters; journalists and other writers; radio and other announcers								
0011	2	Nursing and midwifery professionals and associate professionals								
0012	2	Education methods specialists and inspectors, and unspecified teaching professionals								
0014	2	Social scientists, philosophers and historians								
0015	2	Physiotherapists and unspecified health professionals								
0019	2	Sculptors, painters and commercial designers								
0032	2	Physicists, astronomers and meteorologists								
0033	2	Mathematicians, statisticians and related professionals								
0034	2	Lawyers, judges and other legal professionals								
0037	2	Agronomy and forestry advisers and technicians								
2113	2	Chemists								
2114	2	Geologists and geophysicists								
2130	2	Computing professionals								
2141	2	Architects, town and traffic planners								
2142	2	Civil engineers								
2143	2	Electrical engineers								
2144	2	Electronics and telecommunications engineers					-			
2145	2	Mechanical engineers								
2146	2	Chemical engineers								
2147	2	Mining engineers, metallurgists and related professionals					-			
2148	2	Cartographers and surveyors								
2149	2	Architects, engineers and unspecified related professionals								
2210	2	Agronomists, forestry professionals and related professionals								
2211	2	Biologists, botanists, zoologists and related professionals								
2212	2	Pharmacologists, pathologists and related professionals								
2221	2	Medical doctors								
2222	2	Dentists								
2223	2	Veterinarians								
2310	2	College, university and higher education teaching professionals								
2320	2	Secondary education teaching professionals								
2330	2	Primary education teaching professionals and associate professionals								
2340	2	Special education teaching professionals								
2410	2	Market research, organizational analysts and unspecified business professionals								

International Journal of
Environmental Research and Public Health

2411	2	Accountants								
2431	2	Archivists and curators								
2432	2	Librarians and related information professionals								
2441	2	Economists								
2445	2	Journalists and other writers; radio and other announcers								
2453	2	Composers, musicians and singers		-						
2455	2	Film, stage and related actors and directors								
2460	2	Religious professionals								
2470	2	Public service administrative professionals								
0010	3	Dietician, nutritionists, optometrists, opticians and unspecified health associate professionals								
0013	3	Personnel and careers professionals; employment agents, labour contractors								
0017	3	Appraisers, auctioneers and unspecified associate professionals in finance and sales								
0018	3	Choreographers, dancers, creative and performing artists								
0035	3	Professionals and associate professionals in social work								
0038	3	Customs, tax and related government associate professionals								
3110	3	Chemical and physical science and engineering technicians								
3112	3	Civil engineering technicians								
3113	3	Electrical engineering technicians								
3114	3	Electronics and telecommunications engineering technicians								
3115	3	Mechanical engineering technicians								
3117	3	Mining and metallurgical technicians					-			
3119	3	Unspecified physical and engineering science technicians								
3120	3	Computer assistants, computer equipment operators and related associate professionals								
3130	3	Photographers and image and sound recording equipment operators								
3132	3	Broadcasting and telecommunications equipment operators							-	
3139	3	Unspecified optical, medical and electronic equipment operators			-				-	
3140	3	Air traffic controllers and safety technicians						-		
3141	3	Ships' engineers					-	-	-	-
3142	3	Ships' deck officers and pilots					-	-	-	-
3143	3	Aircraft pilots and related associate professionals					-	-	-	-
3210	3	Life science technicians					-			
3220	3	Medical assistants, hygienists, health and environmental officers								
3225	3	Dental assistants	-	-	-	-				
3227	3	Veterinary assistants	-	-	-					
3228	3	Pharmaceutical assistants								

3320	3	Pre-primary education teaching professionals and associate professionals					-			
3340	3	Teaching associate professionals					-			
3411	3	Securities and finance dealers and brokers								
3412	3	Insurance representatives		-				-		
3413	3	Estate agents								
3415	3	Technical and commercial sales representatives								
3416	3	Buyers								
3421	3	Trade brokers							-	
3422	3	Clearing and forwarding agents								
3429	3	Unspecified business services agents and trade brokers			-				-	
3430	3	Bookkeepers, property managers, and legal, statistical and administrative associate professionals								
3431	3	Administrative secretaries and related associate professionals								
3450	3	Police inspectors and detectives			-	-	-		-	-
3475	3	Athletes, sports persons and related associate professionals								
3480	3	Religious associate professionals								-
0016	4	Travel consultants and travel agency clerks	-							
0020	4	Cashiers, tellers and other counter clerks								
0039	4	Data processors and secretaries								
0040	4	Unspecified personal services workers								
4120	4	Numerical clerks								
4130	4	Stock and production clerks								
4133	4	Transport clerks	-				-			
4142	4	Mail carriers and sorting clerks								
4190	4	Other office clerks								
4222	4	Receptionists and information clerks								
4223	4	Telephone switchboard operators								
0021	5	Travel attendants and guides								
0036	5	Safety and quality inspectors								
5112	5	Transport conductors								
5121	5	Housekeepers and related workers			-					
5122	5	Cooks								
5123	5	Waiters, waitresses and bartenders								
5130	5	Nursing and care assistants								
5131	5	Child-care workers								
5133	5	Home-based personal care workers								

International Journal of
Environmental Research and Public Health

5139	5	Unspecified personal care and related workers		-						
5140	5	Hairdressers, barbers, beauticians and related workers								
5161	5	Fire-fighters					-	-		
5162	5	Police officers								
5163	5	Prison guards								
5169	5	Unspecified protective services workers								
5210	5	Fashion and other models	-	-	-	-	-	-	-	-
5220	5	Shop, stall and market salespersons and demonstrators								
0003	6	Managers of small enterprises in agriculture, hunting, forestry and fishing, and growers								
0022	6	Animal producers								
6130	6	Crop and animal producers								
6140	6	Motorised farm and forestry related workers					-		-	
0023	7	Miners, shotfirers, stone cutters, and related plant operators					-	-	-	
0024	7	Precision and musical instrument makers, jewellery, glass, ceramics and handicraft workers								
0025	7	Tailors, sewers and other related workers								
7120	7	Concrete placers, plasterers, concrete finishers and related workers					-	-	-	-
7121	7	Builders, bricklayers and stonemasons					-		-	-
7129	7	Carpenters and building frame and related trades workers								
7130	7	Electricians					-			
7131	7	Roofers					-	-	-	-
7132	7	Floor layers and tile setters			-		-	-	-	-
7134	7	Insulation workers					-	-	-	-
7135	7	Glaziers					-	-	-	-
7136	7	Plumbers and pipe fitters					-			-
7140	7	Painters, varnishers and related workers								
7150	7	Building structure cleaners and caretakers					-		-	
7210	7	Sheet-metal workers					-		-	-
7211	7	Metal moulders and coremakers					-	-	-	-
7212	7	Welders and flame cutters								
7214	7	Structural-metal preparers and erectors	-	-			-	-	-	-
7215	7	Riggers and cable splicers	-	-		-	-	-	-	-
7216	7	Underwater workers	-	-			-	-	-	-
7220	7	Blacksmiths, tool-makers and related trades workers and operators							-	
7240	7	Electronics mechanics, fitters and servicers							-	
7250	7	Installers, servicers and repairers of telephone and electrical lines					-	-		-

7341	7	Compositors, typesetters, technical illustrators and related workers		-				-		
7411	7	Butchers, fishmongers and related food preparers								
7420	7	Wood treaters, cabinet-makers and related trades workers								-
7440	7	Pelt, leather and shoemaking trades workers	-	-	-		-	-	-	-
0026	8	Metal- and mineral-products machine operators						-		
0027	8	Chemical-products machine operators								
0028	8	Rubber- and plastic-products machine operators								
0029	8	Book- and paper- products machine operators							-	
0030	8	Agricultural or industrial machinery fitters, and mechanical and electrical equipment assemblers								
0041	8	Photographic and printing workers and machine operators								
0042	8	Bakers and confectionery makers and food and beverage tasters								
0043	8	Machine operators of textile, fur and leather								
0044	8	Grocery and beverage machine operators								
8113	8	Mining and mineral-processing-plant operators					-	-		-
8120	8	Metal-processing plant operators								
8130	8	Glass, ceramics, paper and related plant operators					-			
8150	8	Chemical-processing-plant operators								
8160	8	Power-production and related plant operators					-		-	
8240	8	Wood-products machine and plants operators								
8271	8	Meat- and fish-processing-machine operators								
8272	8	Dairy-products machine operators and workers		-						
8311	8	Locomotive engine drivers					-	-	-	
8312	8	Railway brakers, signallers and shunters			-		-	-	-	-
8320	8	Car, taxi, motorcycle and van drivers								
8323	8	Bus and tram drivers								
8324	8	Heavy truck and lorry drivers								
8332	8	Earth-moving and related plant operators					-			
8333	8	Crane, hoist and related plant operators					-		-	-
8334	8	Lifting-truck operators						-	-	
8340	8	Ships' deck crews and related workers					-	-		-
0031	9	Watchpersons, meter readers and related workers								
9110	9	Street vendors and related workers					-			
9130	9	Domestic helpers, cleaners and related workers								
9140	9	Vehicle, window and related cleaners						-		
9151	9	Messengers, package and luggage porters and deliverers								

9160	9	Garbage collectors and related labourers						-		
9210	9	Agricultural, fishery, forestry and related labourers								
9310	9	Mining and construction labourers								
9320	9	Manufacturing and transport labourers								
9330	9	Transport labourers and freight handlers								

International Journal of
Environmental Research and Public Health

MDPI

Table S4. Heatmap of occupational differences in the age-adjusted one-year cumulative incidence of prolonged SA due to musculoskeletal diseases across three Nordic countries among men and women.

Code	Major Occupation	Title	Men			Women		
			FI	NO	SE	FI	NO	SE
0001	1	Managers in computing services, research and development						
0002	1	Managers of wholesale and retail trade and restaurants and hotels						
0004	1	Managers in manufacturing						
0005	1	Managers in construction						
0006	1	Managers in personal care, cleaning and related services	-			-		
0007	1	Managers in storage and communication; business services enterprises, supply and distribution						
0008	1	Managers in unspecified enterprises						
1110	1	Legislators and senior government officials						
1120	1	Senior officials of special-interest organizations						
1210	1	Directors and chief executives						
1221	1	Production and operations managers in agriculture, hunting, forestry and fishing				-		-
1231	1	Finance and administration managers						
1232	1	Personnel and industrial relations managers						
1233	1	Sales and marketing managers						
1234	1	Advertising and public relations managers						
1239	1	Other unspecified specialist managers						
0009	2	Translators and interpreters; journalists and other writers; radio and other announcers						
0011	2	Nursing and midwifery professionals and associate professionals						
0012	2	Education methods specialists and inspectors, and unspecified teaching professionals						
0014	2	Social scientists, philosophers and historians						
0015	2	Physiotherapists and unspecified health professionals						
0019	2	Sculptors, painters and commercial designers						
0032	2	Physicists, astronomers and meteorologists						
0033	2	Mathematicians, statisticians and related professionals						
0034	2	Lawyers, judges and other legal professionals						
0037	2	Agronomy and forestry advisers and technicians						
2113	2	Chemists						
2114	2	Geologists and geophysicists						
2130	2	Computing professionals						
2141	2	Architects, town and traffic planners						

International Journal of
Environmental Research and Public Health

2142	2	Civil engineers							
2143	2	Electrical engineers							
2144	2	Electronics and telecommunications engineers							
2145	2	Mechanical engineers							
2146	2	Chemical engineers							
2147	2	Mining engineers, metallurgists and related professionals							
2148	2	Cartographers and surveyors							
2149	2	Architects, engineers and unspecified related professionals							
2210	2	Agronomists, forestry professionals and related professionals							
2211	2	Biologists, botanists, zoologists and related professionals							
2212	2	Pharmacologists, pathologists and related professionals							
2221	2	Medical doctors							
2222	2	Dentists							
2223	2	Veterinarians	-						
2310	2	College, university and higher education teaching professionals							
2320	2	Secondary education teaching professionals							
2330	2	Primary education teaching professionals and associate professionals							
2340	2	Special education teaching professionals							
2410	2	Market research, organizational analysts and unspecified business professionals							
2411	2	Accountants							
2431	2	Archivists and curators							
2432	2	Librarians and related information professionals							
2441	2	Economists							
2445	2	Journalists and other writers; radio and other announcers							
2453	2	Composers, musicians and singers	-						
2455	2	Film, stage and related actors and directors							
2460	2	Religious professionals							
2470	2	Public service administrative professionals							
0010	3	Dietician, nutritionists, optometrists, opticians and unspecified health associate professionals							
0013	3	Personnel and careers professionals; employment agents, labour contractors							
0017	3	Appraisers, auctioneers and unspecified associate professionals in finance and sales							
0018	3	Choreographers, dancers, creative and performing artists							
0035	3	Professionals and associate professionals in social work							
0038	3	Customs, tax and related government associate professionals							
3110	3	Chemical and physical science and engineering technicians							

International Journal of
Environmental Research and Public Health

3112	3	Civil engineering technicians						
3113	3	Electrical engineering technicians						
3114	3	Electronics and telecommunications engineering technicians						
3115	3	Mechanical engineering technicians						
3117	3	Mining and metallurgical technicians						
3119	3	Unspecified physical and engineering science technicians						
3120	3	Computer assistants, computer equipment operators and related associate professionals						
3130	3	Photographers and image and sound recording equipment operators						
3132	3	Broadcasting and telecommunications equipment operators						
3139	3	Unspecified optical, medical and electronic equipment operators		-				
3140	3	Air traffic controllers and safety technicians				-		
3141	3	Ships' engineers				-	-	-
3142	3	Ships' deck officers and pilots				-	-	-
3143	3	Aircraft pilots and related associate professionals				-	-	-
3210	3	Life science technicians						
3220	3	Medical assistants, hygienists, health and environmental officers						
3225	3	Dental assistants	-	-	-			
3227	3	Veterinary assistants	-	-				
3228	3	Pharmaceutical assistants						
3320	3	Pre-primary education teaching professionals and associate professionals						
3340	3	Teaching associate professionals						
3411	3	Securities and finance dealers and brokers						
3412	3	Insurance representatives	-			-		
3413	3	Estate agents						
3415	3	Technical and commercial sales representatives						
3416	3	Buyers						
3421	3	Trade brokers					-	
3422	3	Clearing and forwarding agents						
3429	3	Unspecified business services agents and trade brokers		-			-	
3430	3	Bookkeepers, property managers, and legal, statistical and administrative associate professionals						
3431	3	Administrative secretaries and related associate professionals						
3450	3	Police inspectors and detectives		-	-		-	
3475	3	Athletes, sports persons and related associate professionals						
3480	3	Religious associate professionals						-
0016	4	Travel consultants and travel agency clerks						

0020	4	Cashiers, tellers and other counter clerks						
0039	4	Data processors and secretaries						
0040	4	Unspecified personal services workers						
4120	4	Numerical clerks						
4130	4	Stock and production clerks						
4133	4	Transport clerks						
4142	4	Mail carriers and sorting clerks						
4190	4	Other office clerks						
4222	4	Receptionists and information clerks						
4223	4	Telephone switchboard operators						
0021	5	Travel attendants and guides						
0036	5	Safety and quality inspectors						
5112	5	Transport conductors						
5121	5	Housekeepers and related workers		-				
5122	5	Cooks						
5123	5	Waiters, waitresses and bartenders						
5130	5	Nursing and care assistants						
5131	5	Child-care workers						
5133	5	Home-based personal care workers						
5139	5	Unspecified personal care and related workers	-					
5140	5	Hairdressers, barbers, beauticians and related workers						
5161	5	Fire-fighters				-		
5162	5	Police officers						
5163	5	Prison guards						
5169	5	Unspecified protective services workers						
5210	5	Fashion and other models	-	-	-	-	-	-
5220	5	Shop, stall and market salespersons and demonstrators						
0003	6	Managers of small enterprises in agriculture, hunting, forestry and fishing, and growers						
0022	6	Animal producers						
6130	6	Crop and animal producers						
6140	6	Motorised farm and forestry related workers					-	
0023	7	Miners, shotfirers, stone cutters, and related plant operators				-	-	
0024	7	Precision and musical instrument makers, jewellery, glass, ceramics and handicraft workers						
0025	7	Tailors, sewers and other related workers						
7120	7	Concrete placers, plasterers, concrete finishers and related workers				-	-	-

7121	7	Builders, bricklayers and stonemasons					-	-
7129	7	Carpenters and building frame and related trades workers						
7130	7	Electricians						
7131	7	Roofers				-	-	-
7132	7	Floor layers and tile setters		-		-	-	-
7134	7	Insulation workers				-	-	-
7135	7	Glaziers				-	-	-
7136	7	Plumbers and pipe fitters						-
7140	7	Painters, varnishers and related workers						
7150	7	Building structure cleaners and caretakers					-	
7210	7	Sheet-metal workers					-	-
7211	7	Metal moulders and coremakers				-	-	-
7212	7	Welders and flame cutters						
7214	7	Structural-metal preparers and erectors	-					
7215	7	Riggers and cable splicers	-		-	-	-	-
7216	7	Underwater workers	-			-	-	-
7220	7	Blacksmiths, tool-makers and related trades workers and operators					-	
7240	7	Electronics mechanics, fitters and servicers					-	
7250	7	Installers, servicers and repairers of telephone and electrical lines				-		-
7341	7	Compositors, typesetters, technical illustrators and related workers	-			-		
7411	7	Butchers, fishmongers and related food preparers						
7420	7	Wood treaters, cabinet-makers and related trades workers						-
7440	7	Pelt, leather and shoemaking trades workers	-	-		-	-	-
0026	8	Metal- and mineral-products machine operators						
0027	8	Chemical-products machine operators						
0028	8	Rubber- and plastic-products machine operators						
0029	8	Book- and paper- products machine operators					-	
0030	8	Agricultural or industrial machinery fitters, and mechanical and electrical equipment assemblers						
0041	8	Photographic and printing workers and machine operators						
0042	8	Bakers and confectionery makers and food and beverage tasters						
0043	8	Machine operators of textile, fur and leather						
0044	8	Grocery and beverage machine operators						
8113	8	Mining and mineral-processing-plant operators				-		-
8120	8	Metal-processing plant operators						
8130	8	Glass, ceramics, paper and related plant operators						

8150	8	Chemical-processing-plant operators						
8160	8	Power-production and related plant operators					-	
8240	8	Wood-products machine and plants operators						
8271	8	Meat- and fish-processing-machine operators						
8272	8	Dairy-products machine operators and workers	-					
8311	8	Locomotive engine drivers				-	-	
8312	8	Railway brakers, signallers and shunters		-		-	-	-
8320	8	Car, taxi, motorcycle and van drivers						
8323	8	Bus and tram drivers						
8324	8	Heavy truck and lorry drivers						
8332	8	Earth-moving and related plant operators						
8333	8	Crane, hoist and related plant operators					-	-
8334	8	Lifting-truck operators				-	-	
8340	8	Ships' deck crews and related workers				-		-
0031	9	Watchpersons, meter readers and related workers						
9110	9	Street vendors and related workers						
9130	9	Domestic helpers, cleaners and related workers						
9140	9	Vehicle, window and related cleaners				-		
9151	9	Messengers, package and luggage porters and deliverers						
9160	9	Garbage collectors and related labourers				-		
9210	9	Agricultural, fishery, forestry and related labourers						
9310	9	Mining and construction labourers						
9320	9	Manufacturing and transport labourers						
9330	9	Transport labourers and freight handlers						

International Journal of *Environmental Research and Public Health*

MDPI

Table S5. Heatmap of occupational differences in the age-adjusted one-year cumulative incidence of prolonged SA due to mental disorders across three Nordic countries among men and women.

Code	Major Occupation	Title	Men			Women		
			FI	NO	SE	FI	NO	SE
0001	1	Managers in computing services, research and development						
0002	1	Managers of wholesale and retail trade and restaurants and hotels						
0004	1	Managers in manufacturing						
0005	1	Managers in construction						
0006	1	Managers in personal care, cleaning and related services	-			-		
0007	1	Managers in storage and communication; business services enterprises, supply and distribution						
0008	1	Managers in unspecified enterprises						
1110	1	Legislators and senior government officials						
1120	1	Senior officials of special-interest organizations						
1210	1	Directors and chief executives						
1221	1	Production and operations managers in agriculture, hunting, forestry and fishing				-		-
1231	1	Finance and administration managers						
1232	1	Personnel and industrial relations managers						
1233	1	Sales and marketing managers						
1234	1	Advertising and public relations managers						
1239	1	Other unspecified specialist managers						
0009	2	Translators and interpreters; journalists and other writers; radio and other announcers						
0011	2	Nursing and midwifery professionals and associate professionals						
0012	2	Education methods specialists and inspectors, and unspecified teaching professionals						
0014	2	Social scientists, philosophers and historians						
0015	2	Physiotherapists and unspecified health professionals						
0019	2	Sculptors, painters and commercial designers						
0032	2	Physicists, astronomers and meteorologists					−1	
0033	2	Mathematicians, statisticians and related professionals						
0034	2	Lawyers, judges and other legal professionals						
0037	2	Agronomy and forestry advisers and technicians						
2113	2	Chemists						
2114	2	Geologists and geophysicists						
2130	2	Computing professionals					−1	
2141	2	Architects, town and traffic planners						

International Journal of
Environmental Research and Public Health

2142	2	Civil engineers						
2143	2	Electrical engineers						
2144	2	Electronics and telecommunications engineers						
2145	2	Mechanical engineers						
2146	2	Chemical engineers						
2147	2	Mining engineers, metallurgists and related professionals						
2148	2	Cartographers and surveyors						
2149	2	Architects, engineers and unspecified related professionals						
2210	2	Agronomists, forestry professionals and related professionals						
2211	2	Biologists, botanists, zoologists and related professionals						
2212	2	Pharmacologists, pathologists and related professionals						
2221	2	Medical doctors						
2222	2	Dentists						
2223	2	Veterinarians						
2310	2	College, university and higher education teaching professionals						
2320	2	Secondary education teaching professionals						
2330	2	Primary education teaching professionals and associate professionals						
2340	2	Special education teaching professionals						
2410	2	Market research, organizational analysts and unspecified business professionals						
2411	2	Accountants						
2431	2	Archivists and curators						
2432	2	Librarians and related information professionals						
2441	2	Economists						
2445	2	Journalists and other writers; radio and other announcers						
2453	2	Composers, musicians and singers	-					
2455	2	Film, stage and related actors and directors						
2460	2	Religious professionals						
2470	2	Public service administrative professionals						
0010	3	Dietician, nutritionists, optometrists, opticians and unspecified health associate professionals						
0013	3	Personnel and careers professionals; employment agents, labour contractors						
0017	3	Appraisers, auctioneers and unspecified associate professionals in finance and sales						
0018	3	Choreographers, dancers, creative and performing artists						
0035	3	Professionals and associate professionals in social work						
0038	3	Customs, tax and related government associate professionals						
3110	3	Chemical and physical science and engineering technicians						

3112	3	Civil engineering technicians						
3113	3	Electrical engineering technicians						
3114	3	Electronics and telecommunications engineering technicians						
3115	3	Mechanical engineering technicians						
3117	3	Mining and metallurgical technicians						
3119	3	Unspecified physical and engineering science technicians						
3120	3	Computer assistants, computer equipment operators and related associate professionals						
3130	3	Photographers and image and sound recording equipment operators						
3132	3	Broadcasting and telecommunications equipment operators						
3139	3	Unspecified optical, medical and electronic equipment operators						
3140	3	Air traffic controllers and safety technicians				-		
3141	3	Ships' engineers				-		-
3142	3	Ships' deck officers and pilots				-		-
3143	3	Aircraft pilots and related associate professionals				-		-
3210	3	Life science technicians						
3220	3	Medical assistants, hygienists, health and environmental officers						
3225	3	Dental assistants	-		-			
3227	3	Veterinary assistants	-					
3228	3	Pharmaceutical assistants						
3320	3	Pre-primary education teaching professionals and associate professionals						
3340	3	Teaching associate professionals						
3411	3	Securities and finance dealers and brokers						
3412	3	Insurance representatives	-			-		
3413	3	Estate agents						
3415	3	Technical and commercial sales representatives						
3416	3	Buyers						
3421	3	Trade brokers						
3422	3	Clearing and forwarding agents						
3429	3	Unspecified business services agents and trade brokers						
3430	3	Bookkeepers, property managers, and legal, statistical and administrative associate professionals					−1	
3431	3	Administrative secretaries and related associate professionals		−1			−1	
3450	3	Police inspectors and detectives			-			-
3475	3	Athletes, sports persons and related associate professionals					−1	
3480	3	Religious associate professionals					−1	-
0016	4	Travel consultants and travel agency clerks					−1	

International Journal of
Environmental Research and Public Health

0020	4	Cashiers, tellers and other counter clerks						
0039	4	Data processors and secretaries						
0040	4	Unspecified personal services workers		−1				
4120	4	Numerical clerks		−1				
4130	4	Stock and production clerks						
4133	4	Transport clerks						
4142	4	Mail carriers and sorting clerks						
4190	4	Other office clerks						
4222	4	Receptionists and information clerks						
4223	4	Telephone switchboard operators						
0021	5	Travel attendants and guides						
0036	5	Safety and quality inspectors						
5112	5	Transport conductors					−1	
5121	5	Housekeepers and related workers						
5122	5	Cooks		−1			−1	
5123	5	Waiters, waitresses and bartenders						
5130	5	Nursing and care assistants						
5131	5	Child-care workers		-1			−1	
5133	5	Home-based personal care workers						
5139	5	Unspecified personal care and related workers	-					
5140	5	Hairdressers, barbers, beauticians and related workers						
5161	5	Fire-fighters				-		
5162	5	Police officers						
5163	5	Prison guards						
5169	5	Unspecified protective services workers						
5210	5	Fashion and other models	-			-		-
5220	5	Shop, stall and market salespersons and demonstrators						
0003	6	Managers of small enterprises in agriculture, hunting, forestry and fishing, and growers						
0022	6	Animal producers		−1				
6130	6	Crop and animal producers						
6140	6	Motorised farm and forestry related workers						
0023	7	Miners, shotfirers, stone cutters, and related plant operators				-		
0024	7	Precision and musical instrument makers, jewellery, glass, ceramics and handicraft workers						
0025	7	Tailors, sewers and other related workers						
7120	7	Concrete placers, plasterers, concrete finishers and related workers				-		-

7121	7	Builders, bricklayers and stonemasons						-
7129	7	Carpenters and building frame and related trades workers						
7130	7	Electricians						
7131	7	Roofers				-		-
7132	7	Floor layers and tile setters				-		-
7134	7	Insulation workers		−1		-	−1	-
7135	7	Glaziers				-		-
7136	7	Plumbers and pipe fitters						-
7140	7	Painters, varnishers and related workers					−1	
7150	7	Building structure cleaners and caretakers					−1	
7210	7	Sheet-metal workers					−1	-
7211	7	Metal moulders and coremakers				-		-
7212	7	Welders and flame cutters						
7214	7	Structural-metal preparers and erectors	-				−1	
7215	7	Riggers and cable splicers	-	−1	-	-	−1	-
7216	7	Underwater workers	-			-	−1	-
7220	7	Blacksmiths, tool-makers and related trades workers and operators					−1	
7240	7	Electronics mechanics, fitters and servicers						
7250	7	Installers, servicers and repairers of telephone and electrical lines				-		-
7341	7	Compositors, typesetters, technical illustrators and related workers	-			-	−1	
7411	7	Butchers, fishmongers and related food preparers					−1	
7420	7	Wood treaters, cabinet-makers and related trades workers					−1	-
7440	7	Pelt, leather and shoemaking trades workers	-			-		-
0026	8	Metal- and mineral-products machine operators				-		
0027	8	Chemical-products machine operators					−1	
0028	8	Rubber- and plastic-products machine operators					−1	
0029	8	Book- and paper- products machine operators					−1	
0030	8	Agricultural or industrial machinery fitters, and mechanical and electrical equipment assemblers					−1	
0041	8	Photographic and printing workers and machine operators						
0042	8	Bakers and confectionery makers and food and beverage tasters						
0043	8	Machine operators of textile, fur and leather						
0044	8	Grocery and beverage machine operators						
8113	8	Mining and mineral-processing-plant operators		−1		-	−1	-
8120	8	Metal-processing plant operators						
8130	8	Glass, ceramics, paper and related plant operators						

8150	8	Chemical-processing-plant operators						
8160	8	Power-production and related plant operators						
8240	8	Wood-products machine and plants operators					−1	
8271	8	Meat- and fish-processing-machine operators						
8272	8	Dairy-products machine operators and workers	-					
8311	8	Locomotive engine drivers				-		
8312	8	Railway brakers, signallers and shunters				-	−1	-
8320	8	Car, taxi, motorcycle and van drivers		−1			−1	
8323	8	Bus and tram drivers						
8324	8	Heavy truck and lorry drivers						
8332	8	Earth-moving and related plant operators						
8333	8	Crane, hoist and related plant operators						-
8334	8	Lifting-truck operators				-	−1	
8340	8	Ships' deck crews and related workers				-	−1	-
0031	9	Watchpersons, meter readers and related workers						
9110	9	Street vendors and related workers						
9130	9	Domestic helpers, cleaners and related workers						
9140	9	Vehicle, window and related cleaners				-		
9151	9	Messengers, package and luggage porters and deliverers						
9160	9	Garbage collectors and related labourers				-		
9210	9	Agricultural, fishery, forestry and related labourers						
9310	9	Mining and construction labourers						
9320	9	Manufacturing and transport labourers						
9330	9	Transport labourers and freight handlers						
